# Graphene-based metasurface solar absorber design with absorption prediction using machine learning

**DOI:** 10.1038/s41598-022-06687-6

**Published:** 2022-02-16

**Authors:** Juveriya Parmar, Shobhit K. Patel, Vijay Katkar

**Affiliations:** 1 Department of Electronics and Communication, Marwadi University, Rajkot, 360003 India; 2 Department of Computer Engineering, Marwadi University, Rajkot, 360003 India

**Keywords:** Metamaterials, Optical sensors, Solar energy and photovoltaic technology

## Abstract

Solar absorber is required to absorb most of the energy of the solar spectral irradiance. We propose a graphene-based solar absorber design with two different metasurfaces to improve this absorption and increase the efficiency of the solar absorber. The metasurfaces are selected based on their symmetrical/asymmetrical nature (O-shape and L-shape). The O-shape metasurface design is showing better performance over the L-shape metasurface design. The absorption performance is also compared with AM 1.5 solar spectral irradiance to show the effectiveness of the solar absorber. The absorption values are also enhanced by varying the parameters like resonator thickness and substrate thickness. The proposed solar absorber design gives maximum absorption in the ultraviolet and visible range. Furthermore, the design is also showing a high and similar absorption rate over a wide angle of incidence. The absorption of O-shape metasurface design is also predicted using machine learning. 1D-Convolutional Neural Network Regression is used to develop a Machine Learning model to determine absorption values of intermediate wavelength for assorted values of angle of incidence, resonator thickness, and substrate thickness. The results of experiments reveal that absorption values may be predicted with a high degree of accuracy. The proposed absorber with its high absorbing capacity can be applied for green energy applications.

## Introduction

Energy demand is increasing day by day because of rapid industrialization and an increase in household energy consumption. Renewable and clean energy is the ultimate solution for these increasing energy demands as it is not affecting the environment. Solar energy is one of the best clean, renewable natural sources because of its ease of availability. It can be one of the best ways to cope up with the increasing energy demand. Still to make the solar energy utilization more efficient. Solar energy can also be easily converted into other forms of energy which can be applied for the designing purpose of solar cells, photovoltaic devices, and solar absorbers, etc. A perfect absorber is quietly prudent for various applications consisting of solar energy harvesting, light modulation, and sensing, etc.^1–6^. Metamaterials have attracted a lot of attention and they are the unique candidate due to their unique electro-optical characteristics and compact size rather than bulky optical devices^7–12^. Due to this, they have various applications comprising from polarizers, detectors to absorbers^13–17^ each working at various operating frequencies, i.e., microwave, terahertz, ultraviolet, visible, infrared, etc.^18–22^.

Graphene is also an essential element for the advancement of physics, photonics-like fields. From the available research on graphene, we can say that graphene is a two-dimensional element with an astonishing electro-crystal characteristic that plays a major role in designing applications that are based on recent physics and other concerned domains. To conclude, graphene is a new group of elements that is only an atom-thick but has helped to obtain the results in the subdomain of physics known as low magnitude physics^23^. Graphene has remarkable electro-optical, thermal, mechanical properties which can be applied with a solar absorber^24^. As per the research, filtered graphene sheet is highly transparent and flexible because graphene’s every single layer with refractivity of 0.1% absorbs up to 2.3% of white light^25^. Studies also indicate that as the no. of graphene sheet increases so does the absorption rate^26^.

Rifat et al. reported a near-perfect solar absorber that achieved 99.8% absorption at 932 nm wavelength^27^. Yu et al. presented a solar absorber design based on refractory metal which obtained around 90% absorption in the wide range of visible and near-infrared (NIR) range of 360 to 1600 nm^28^. Patel and co-authors presented a highly efficient terahertz range which obtained 89.57%, 97.5%, and 92.7% absorption in the ultraviolet, visible, and overall solar range^29^. Al-RJoub et al. fabricated a solar absorber design with high-temperature application in mind which achieved an absorption of 95%^30^. Wu and co-authors presented a metamaterial-based near-perfect and ultra-broadband solar absorber that achieved around 95% for the 400 to 2000 nm range of visible and NIR^31^. Liu et al. reported a solar absorber that achieved around 90% absorption in the range of 316 to 1400 nm comprising visible and NIR ranges^32^. Bilal et al. presented a tungsten nanowire consisting of a solar absorber design which obtained near-unity absorption in the ultraviolet region but only 85% absorption in visible range^33^.

In this paper, we have proposed a high absorption graphene-based absorber that is broadband for the ultraviolet and visible range. We have also carried out a detailed comparative analysis of the o-shape metasurface design that gives better absorption by varying the different physical parameters of the design. The second section presents the graphene-based metasurface design, the third section presents parametric analysis and the fourth section presents the design of 1D-Convolutional Neural Network (CNN) regression model to predict the absorption values of intermediate wavelengths and corresponding experimental results. The concluding remarks are presented in the fifth section.

## Graphene-based metasurface design

**Figure 1:**
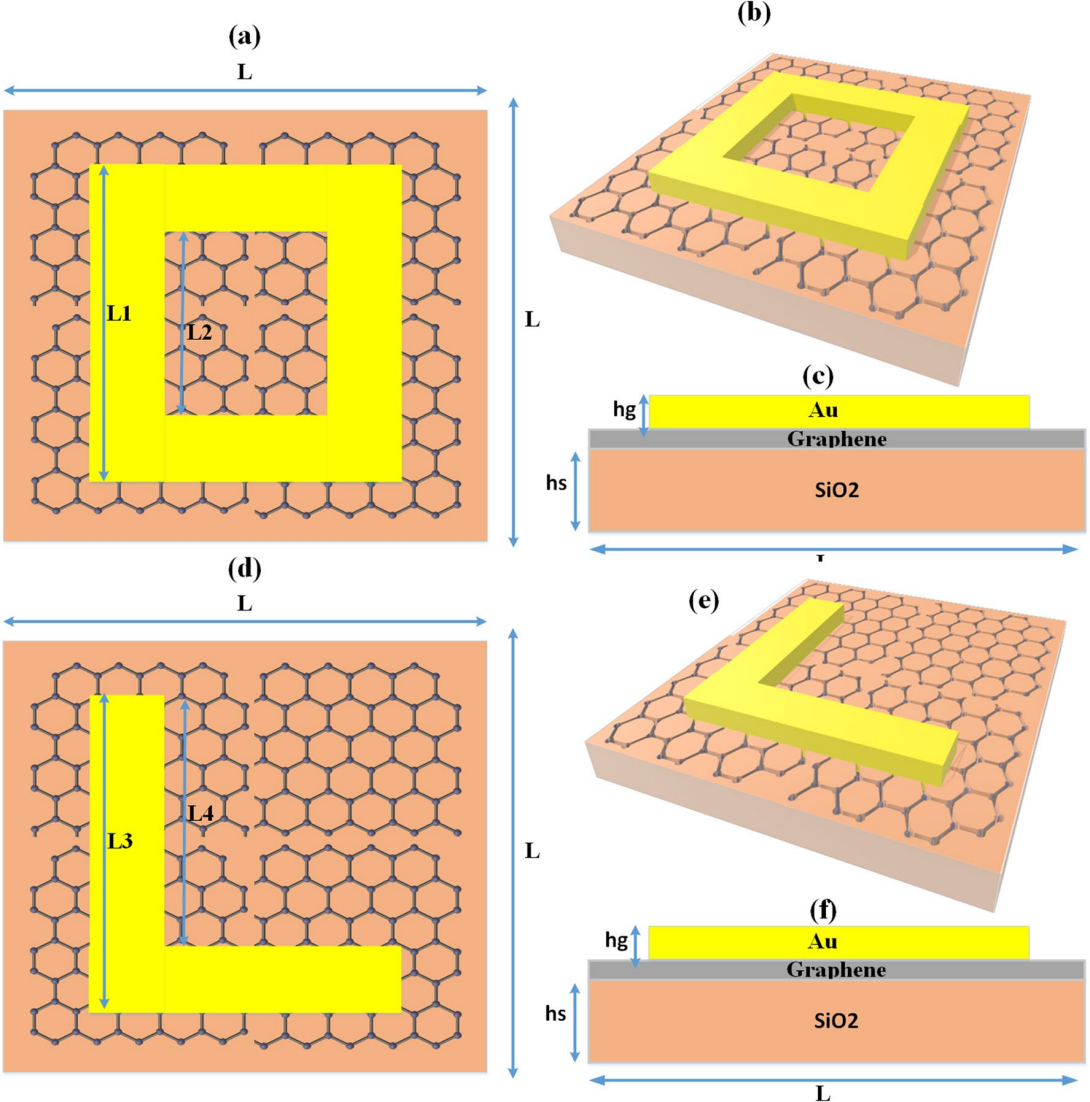
Graphene-based metasurface design (**a**) O-shape metasurface design top view having size L1 = 2.4 µm, L2 = 1 µm (**b**) O-shape metasurface design 3D view (**c**) O-shape metasurface design front view (**d**) O-shape metasurface design top view having size L3 = 2.4 µm, L4 = 2.2 µm (**e**) O-shape metasurface design 3D view (**f**) O-shape metasurface design front view. The overall area of the structure 3 × 3 µm^2^ The dimensions are not up to the scale.

Graphene-based metasurface absorber design is presented in Fig. 1 for two different metasurfaces (O-shape metasurface and L-shape metasurface). The reason for selecting these two shapes is because one is symmetrical design and the other one is asymmetrical design. The design is made with a gold resonator based on SiO_2_ substrate with graphene monolayer as a spacer sandwiched between the two layers. The two different metasurfaces are created by changing the shape of the metasurface as presented in their top views (Fig. 1a,d). The side views and 3d views are also presented in the schematic. The light is falling on the top of the resonator which is an O-shape and L-shape for two metasurface absorbers. The O-shape metasurface absorber is increasing the inductance by connected resonator shape and it is also symmetrical. The L-shape metasurface is having a change in inductance and capacitance compared to the O-shape metasurface as there is no connected path like the O-shape metasurface. The L-shape is also not symmetric which gives rise to the polarization sensitiveness^34^ compared to the symmetric shape of the O-shape metasurface which is polarization insensitive. The metasurface absorber structure presented in Fig. 1 is a periodic structure with a period of 3 µm in both directions. The different geometrical parameter values presented in Fig. 1 are L: 3 µm, L1: 2.4 µm, L2: 1 µm, L3: 2.4 µm, L4: 2.2 µm hs: 1 µm; hg: 0.6 µm and the graphene monolayer thickness is 0.34 nm. The use of graphene material is essential in this design because the graphene with its high conductivity contributes to enhancing the absorption and achieving the broadband design. The conductivity (${\sigma }_{s}$) of graphene depends on graphene chemical potential (${\mu }_{c}$) and is presented in Eqs. (1–4).

1 $$\varepsilon \left(\omega \right)=1+\frac{{\sigma }_{s}}{{\varepsilon }_{0}\omega \Delta },$$

2 $${\sigma }_{intra}=\frac{-j{e}^{2}{k}_{B}T}{\pi {\hslash }^{2}(\omega -j2\Gamma )}\left(\frac{{\mu }_{c}}{{k}_{B}T}+2\mathit{ln}\left({e}^{-\frac{{\mu }_{c}}{{k}_{B}T}}+1\right)\right),$$

3 $${\sigma }_{inter}= \frac{-j{e}^{2}}{4\pi \hslash }\mathit{ln}\left(\frac{2\left|{\mu }_{c}\right|-(\omega -j2\Gamma )\hslash }{2\left|{\mu }_{c}\right|+(\omega -j2\Gamma )\hslash }\right),$$

4 $${\sigma }_{s}={\sigma }_{inter}+{\sigma }_{intra}.$$

The different parameters used in the equations are: scattering rate (Γ), Reduced plank constant ($\mathrm{\hslash }$), Boltzmann constant (k_B_), Electron relaxation time (τ^−1^) and $\Delta$ is the thickness of graphene which is 0.34 nm. The fabrication of graphene monolayer sheets can be done with chemical vapour deposition (CVD)^35^ or cleavage technique^36^ or lithography^37^.

**Figure 2:**
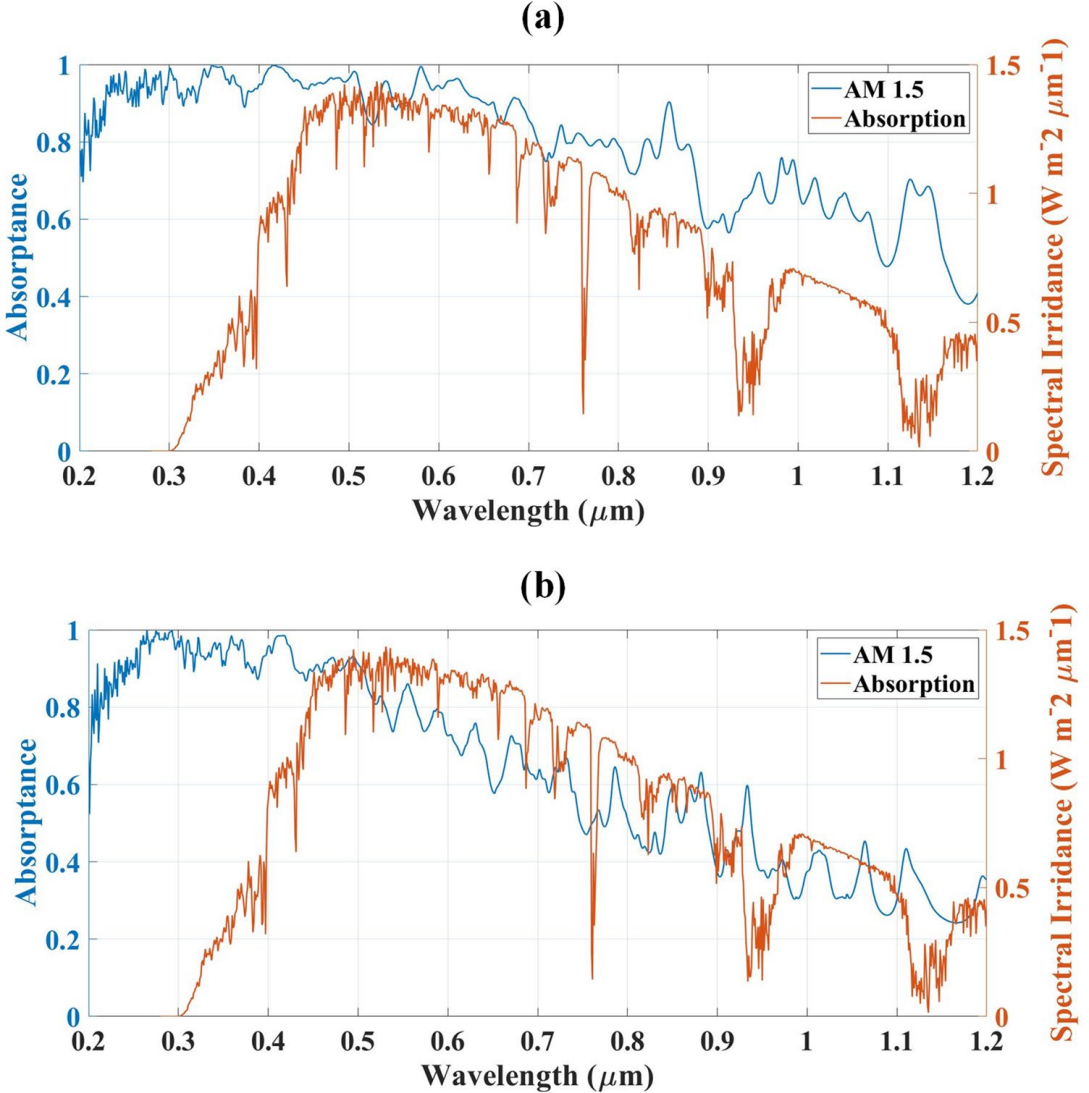
Absorption response comparison with AM1.5 specral irridance for (**a**) O-shape metasurface absorber design (**b**) L-shape metasurface absorber design. The O-shape metasurface absorber is having better absorption for all the regions.

The metasurface designs are simulated using the Finite Element Method (FEM) based COMSOL Simulator and the results in the form of absorption are presented and compared^38–46^ in Fig. 2 and Table 1. The light is falling from the top of the resonator structure and periodic boundary conditions are used for the simulations. The meshing condition of the structure is set as tetrahedral Delaunay tessellation. The absorption response for O-shape metasurface absorber design and L-shape metasurface absorber design is presented in Fig. 2a,b respectively. The blue colour region shows the absorption plot and its values on the left-hand side for our proposed design and the orange colour shows the AM 1.5 spectral irradiance plot with its values on the right-hand side. The absorption response is also compared with AM 1.5 spectral irradiance. The comparison with irradiance shows the effectiveness of the absorber as more absorption in high irradiance shows the higher efficiency of the absorber. The response shows that the O-shape metasurface absorber design is showing near to unity absorption for the high spectral irradiance in the visible region which makes the O-shape metasurface more effective in solar energy harvesting applications. The L-shape metasurface absorber is having less absorption for the high spectral irradiance region as presented in Fig. 2b.

**Table 1:** Comparison of solar absorber designs.

Designs	0.2 to 0.4 µm (ultraviolet region) Average absorption (%)	0.4 to 0.7 µm (visible region) Average absorption (%)
O-shaped metasurface absorber design	91	94
L-shaped metasurface absorber design	90	83
Ref.^38^	90	90
Ref.^39^	-	93.7
Ref.^40^	–	93
Ref.^41^	–	92
Ref.^42^	–	90
Ref.^43^	–	86.5
Ref.^44^	–	80
Ref.^45^	–	71.1
Ref.^46^	–	70
Ref.^28^	–	90

**Figure 3:**
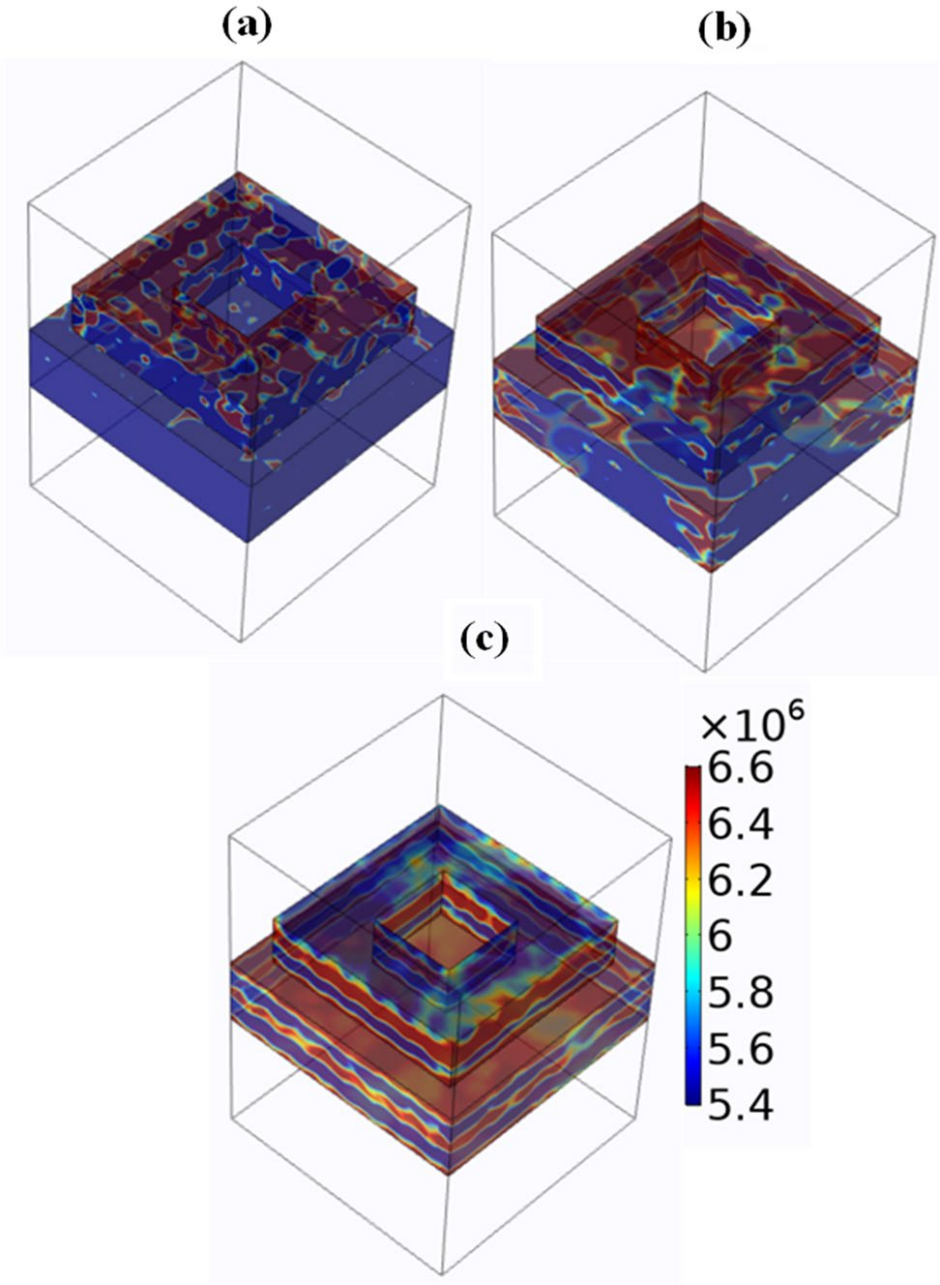
Electric field intensity (V/m) of graphene-based O-shape metasurface absorber for wavelengths (**a**) 0.5 µm (**b**) 0.8 µm (**c**) 1.2 µm. The color bar indicates color values of electric field intensity in V/m. Three different wavelengths are selected to show the overall absorption for visible and near infrared regions.

We have also analyzed the electric field for O-shape metasurface design (Fig. 3) to observe and verify the absorption in terms of the electric field. The selection of three different wavelengths is carried out to observe the electric field at different absorption levels. The three wavelengths selected are 0.5 µm, 0.8 µm, and 1.2 µm. The results clearly show that for the initial wavelength the electric field is more around the resonator and as the wavelength increases the absorption increases in the substrate. The electric field is measured in V/m and its values are given in a scale on the right-hand side of Fig. 3. The red colour indicated the high electric field (6.6 × 10^6^ V/m).

## Parametric analysis

**Figure 4:**
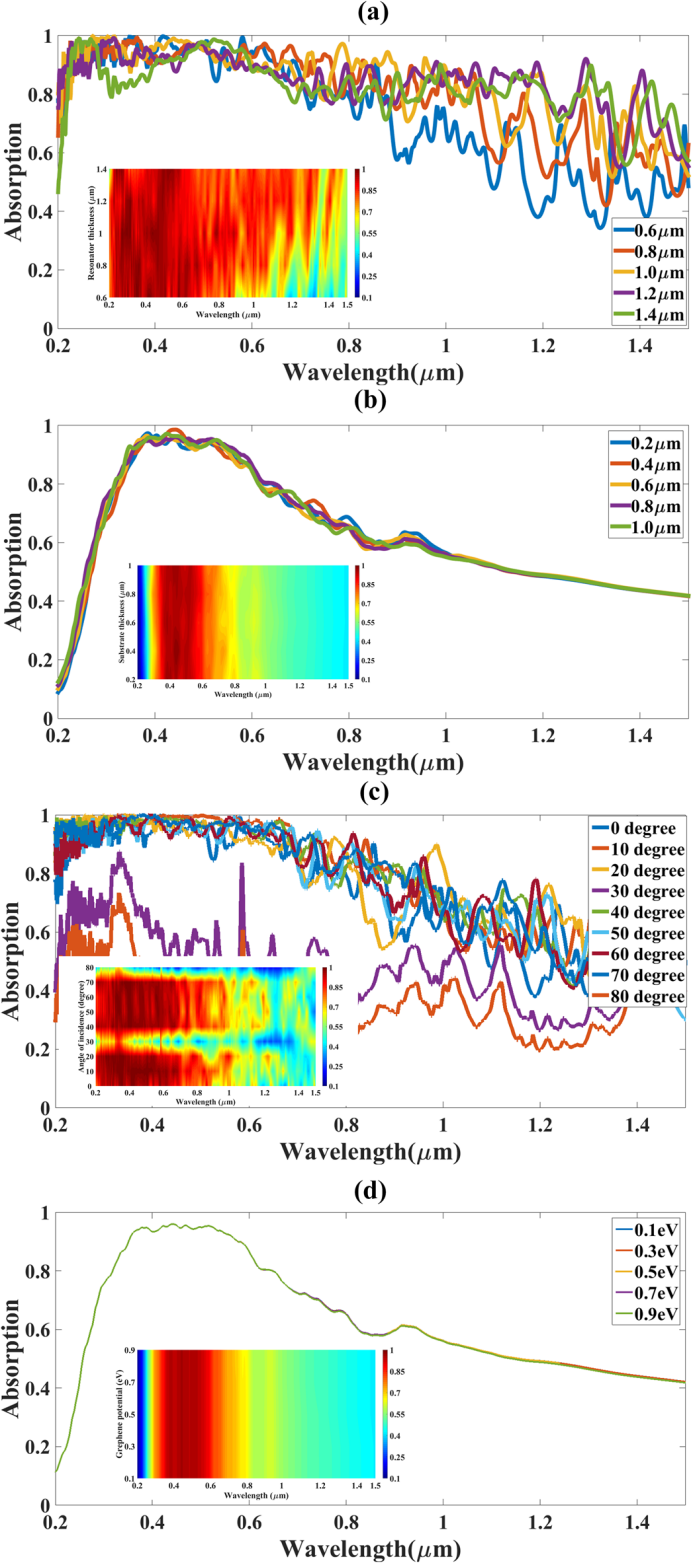
Absorption response of graphene-based O-shape metasurface absorber for (**a**) Different resonator thicknesses (0.6 to 1.4 µm). (**b**) Different substrate thicknesses (0.2 to 1 µm). (**c**) Difference angles of incidence (0° to 80°). (**d**) Different graphene’s chemical potential (0.1 to 0.9 eV. The inset shows the color plot of absorption for different resonator/substrate thickness/angle of absorption/graphene chemical potential for the wavelength range of 0.2 to 1.5 µm.

The absorption of the absorber is dependent on its geometrical parameters so we have applied the parametric analysis of two geometrical parameters namely substrate thickness and resonator thickness (Fig. 4a,b). We have also varied the angle of incidence and graphene chemical potential (Fig. 4c,d) to observe its effect on absorption. The analysis is presented for the 0.2 to 1.2 µm wavelength range. The resonator thickness is varied for 0.6 to 1.4 µm and substrate thickness is varied from 0.2 to 1 µm and absorption results are presented in Fig. 4a,b respectively. The colour plot for the variation is presented as an inset in the figure for a better understanding of the results. The variation in resonator thickness clearly shows that for 0.6 µm value, the absorption is highest in overall best with more than 92% average absorption in visible and ultraviolet regions. As we increase the resonator thickness greater than 0.6 µm, the absorption decreases in the visible and ultraviolet region but increase in the infrared region because the inductance effect is increased as the thickness of the resonator is increasing which results in a change in absorption in higher wavelength. The substrate thickness is varied from 0.2 to 1 µm and the results clearly show that as the thickness is increased, the absorption is increasing and the best values are available for 1 µm thickness. The reason for the increase in absorption is because the higher thickness increases the area of the substrate, which increases the absorption in the substrate. The resonator thickness of 0.6 µm and substrate thickness is 1 µm is fixed after this parametric analysis.

The graphene chemical potential is varied from 0.1 to 0.9 eV and we are getting similar absorption for all the variation which clearly show that at visible and ultraviolet wavelength range the effect of graphene’s chemical potential is minimum. The angle of incidence is very important for designing solar absorbers so we have also varied the angle of incidence from 0° to 80° and the results clearly show that we are getting a wide-angle of incidence and the absorption is almost the same for all angles except 30° and 80°. The results clearly show that for angle of incidence 30° and 80°, the average absorption is more than 60% and for the rest of the angles it is more than 92%. These parameter variations clearly show that graphene chemical potential has minimum effect in absorption response while the wide-angle of incidence is also achieved for the angle of incidence variation of light falling on the structure.

## Machine learning (1D-CNN regression model for prediction)

Machine learning is used to predict the absorption values for intermediate/missing wavelengths so that simulation and experimentation time can be reduced. 1D-Convolutional Neural Network (1D-CNN) in comparison to Multilayer Perceptron Regression and other traditional machine learning regression algorithms, can capture the complicated relationship between two parameters/variables with more minute details^47^. Thus, experiments are performed using 1D-CNN Regression to build the model for predicting absorption values.

**Figure 5:**
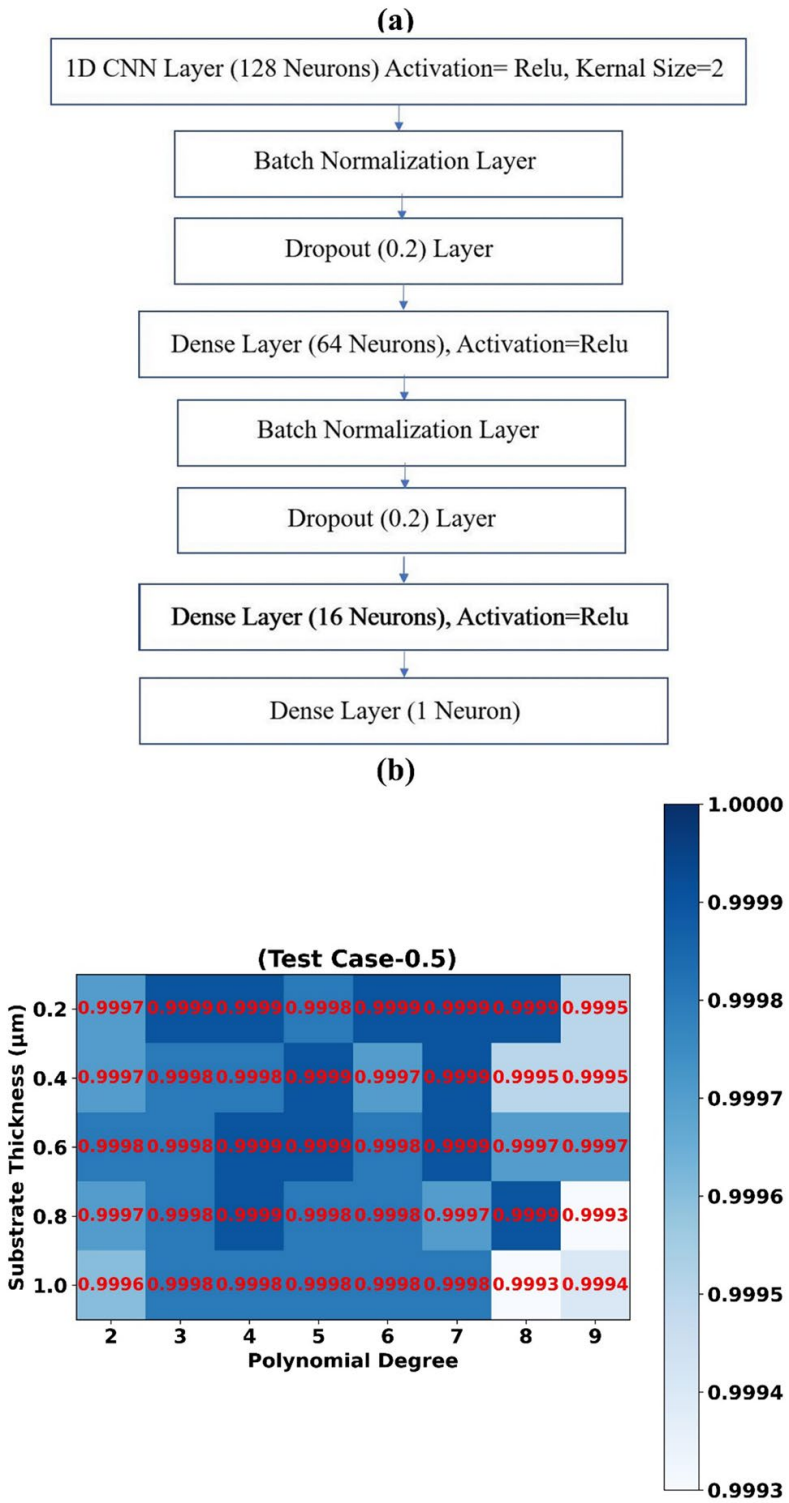
(**a**) Layer structure of 1D-CNN regressor (**b**) Prediction accuracy (R^2^ Score) of 1D-CNN Regressor models trained for assorted values of substrate thickness and polynomial degree features (Test Case C-0.5).

Layer structure of 1D-CNN Regression model is depicted in Fig. 5a. First Layer contains 128 neurons with kernel size of 2. Layer 2, layer 5 are batch normalization layers. Layer 3 and layer 5 are dropout layers that prevent models from overfitting. Layer 4, layer 7 contains 64, 16 neurons respectively. Layer 8 is single neuron output layer. Relu activation function is used in layer1 and layer 4. 1D-CNN Regression model is trained using 1000 epochs. Machine Learning models are built using Python 3.8, scikit-learn library version 1.0, and tensorflow library version 2.7.

Four Test Cases (T.C.-0.2, T.C.-0.3, T.C.-0.4, T.C.-0.5) are used to test the prediction accuracy. Two non-overlapping subsets of data are generated by randomly selecting 20%, 30%, 40%, 50% records from simulated data in Test cases T.C.-0.2, T.C.-0.3, T.C.-0.4, and T.C.-0.5 respectively. Randomly selected records are used to evaluate the performance of Machine Learning model which is trained using remaining records of simulated data. R^2^ score is used as a metric to evaluate the prediction accuracy of regression models. R^2^ score is calculated using Eq. (5).

5 $${R}^{2}= 1- \frac{{SS}_{red}}{{SS}_{tot}},$$

6 $${SS}_{tot}=\sum_{i=1}^{N}{({ActualTarget}_{i}- Average\, TargetValue )}^{2},$$

7 $${SS}_{res}={\sum }_{1}^{N}{({Predicted\, TargetValue}_{i}-{ActualTargetValue}_{i})}^{2}.$$

Here, SS_res_ is the sum of squares of the residual errors and SS_tot_ is the total sum of the errors and N is the number of testing records.

All R^2^ score values are rounded off to four decimal places while representing the results. Reason for using higher degree polynomial features for training 1D-CNN regressor models is explained in supplementary material with the help of Supplementary Fig. S1.

### Substrate thickness

R^2^ scores obtained by trained 1D-CNN Regression models (Test Case-0.5) for assorted combinations of substrate thickness values with polynomial degree features are depicted using heat map in Fig. 5b. Highest R^2^ score of 0.9999, 0.9999, 0.9999, 0.9999, 0.9998 is achieved for substrate thickness value 0.2 µm, 0.4 µm, 0.6 µm, 0.8 µm, 1.0 µm respectively. Scatter plots of predicted absorption values by different 1D-CNN Regression models vs actual absorption values for test case-0.5 and substrate thickness 0.2 µm, 0.4 µm, 0.6 µm are shown in Fig. 7a–c respectively.

R^2^ scores obtained by trained 1D-CNN Regression models for Test Case-0.4, Test Case-0.3, Test Case-0.2, and assorted combinations of substrate thickness values with polynomial degree features are depicted using heat map in Supplementary Fig. S2a–c. Scatter plots of predicted absorption values by different 1D-CNN Regression models vs actual absorption values for test case-0.5 and substrate thickness 0.8 µm, 1.0 µm are shown in Supplementary Fig. S5a,b respectively. Similarly for test case-0.4 and substrate thickness 0.2 µm, 0.4 µm, 0.6 µm, 0.8 µm, 1.0 µm scatter plots are shown in Supplementary Figs. S6a–c, S7a,b respectively.

### Resonator thickness

**Figure 6:**
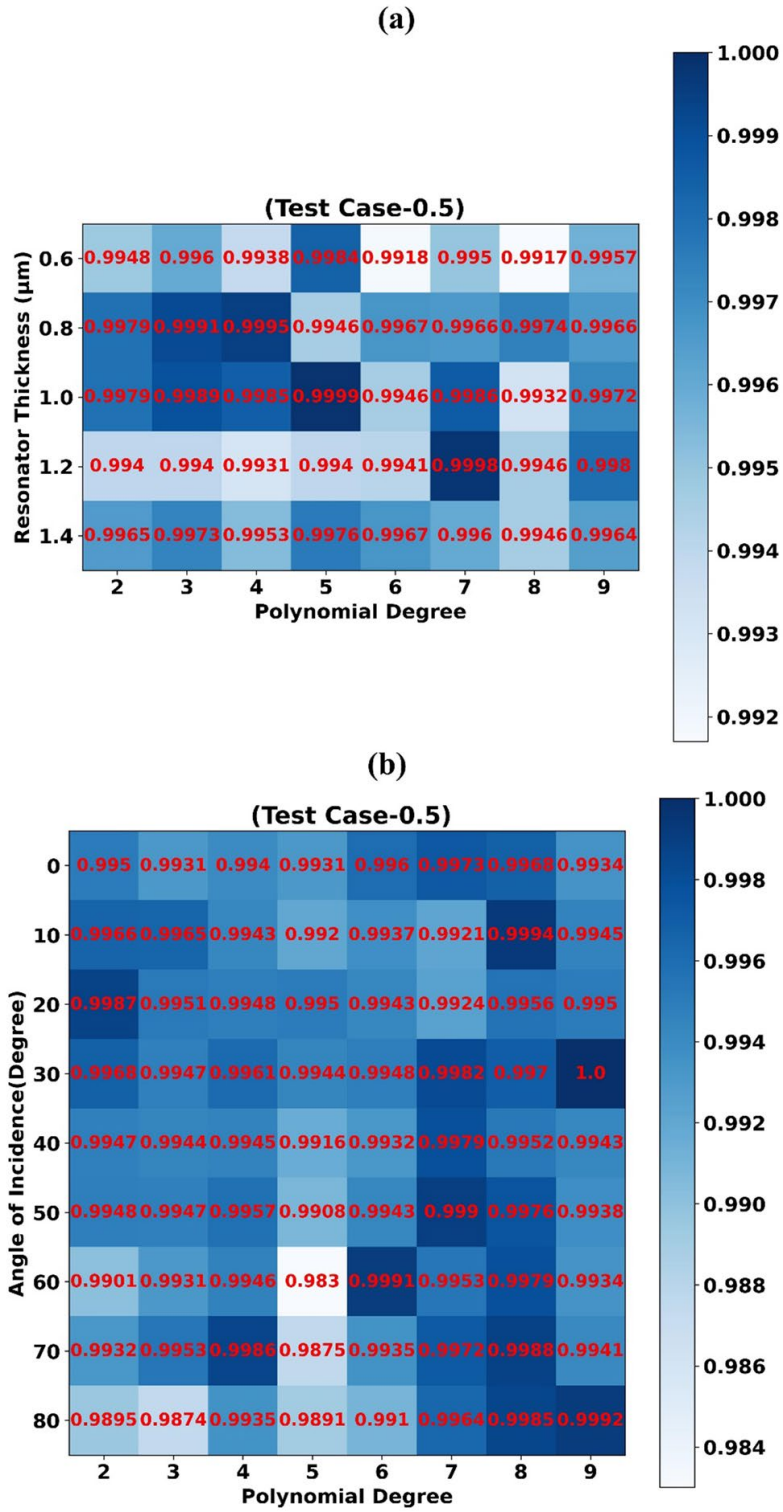
Prediction accuracy (R^2^ Score) of 1D-CNN Regressor models trained for assorted values of (**a**) Resonator thickness and polynomial degree features (Test Case C-0.5) (**b**) Angle of incidence and polynomial degree features (Test Case C-0.5).

**Figure 7:**
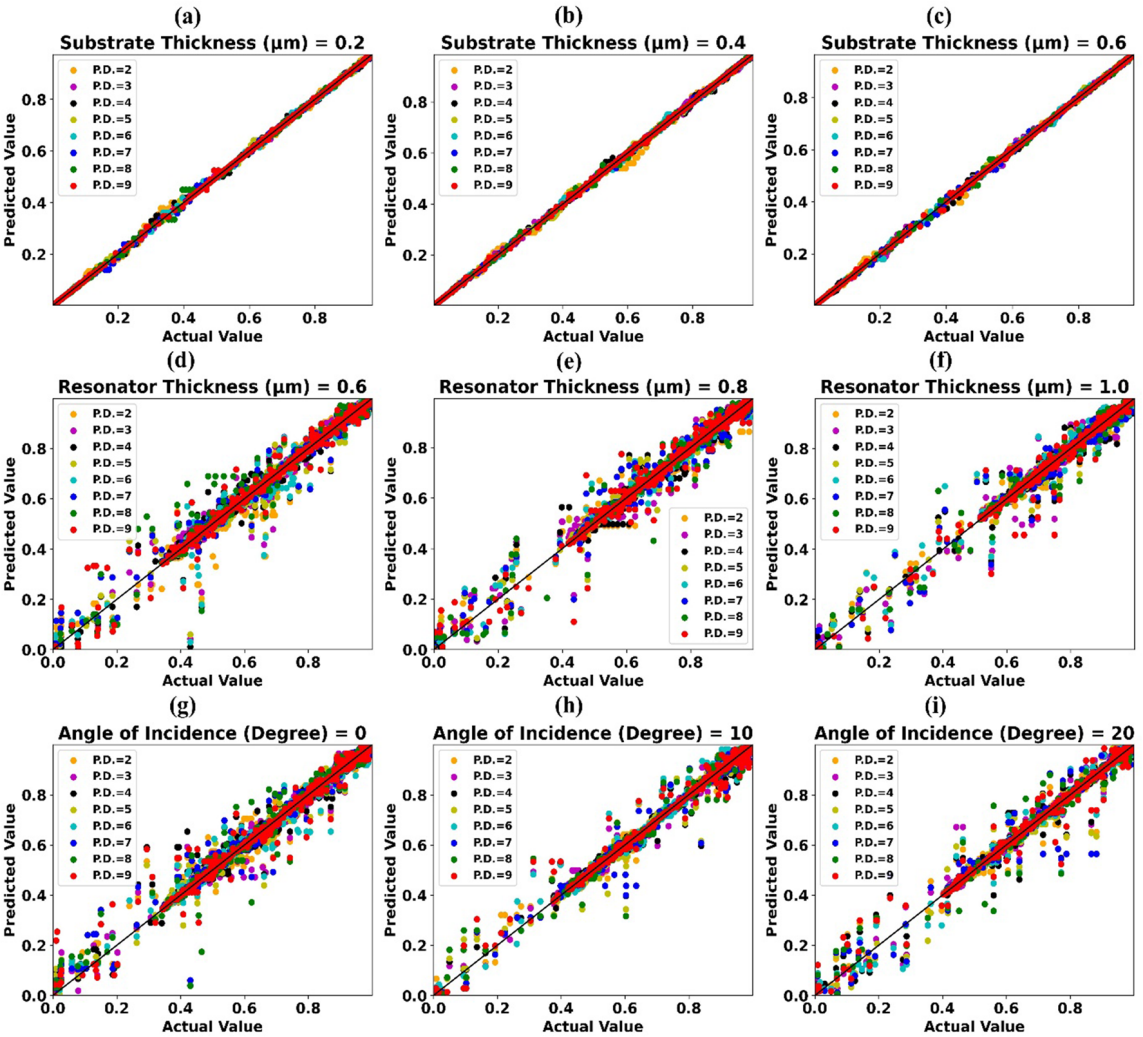
Predicted absorption value by different 1D CNN Regressors vs Actual absorption value for test case C-0.5 and (**a**) Substrate thickness = 0.2 µm (**b**) Substrate thickness = 0.4 µm (**c**) Substrate thickness = 0.6 µm (**d**) Resonator thickness = 0.6 µm (**e**) Resonator thickness = 0.8 µm (**f**) Resonator thickness = 1.0 µm (**g**) Angle of incidence = 0° (**h**) Angle of incidence = 10° (**i**) Angle of incidence = 20°.

R^2^ scores obtained by trained 1D-CNN Regression models (Test Case-0.5) for assorted combinations of resonator thickness values with polynomial degree features are depicted using heat map in Fig. 6a. Highest R^2^ score of 0.9984, 0.9995, 0.9999, 0.9998, 0.9976 is achieved for resonator thickness value 0.6 µm, 0.8 µm, 1.0 µm, 1.2 µm, 1.4 µm respectively. Scatter plots of predicted absorption values by different 1D-CNN Regression models vs actual absorption values for test case-0.5 and resonator thickness 0.6 µm, 0.8 µm, 1.0 µm are shown in Fig. 7d–f respectively.

R^2^ scores obtained by trained 1D-CNN Regression models for Test Case-0.4, Test Case-0.3, Test Case-0.2, and assorted combinations of resonator thickness values with polynomial degree features are depicted using heat map in Supplementary Fig. S3a–c. Scatter plots of predicted absorption values by different 1D-CNN Regression models vs actual absorption values for test case-0.5 and resonator thickness 1.2 µm, 1.4 µm are shown in Supplementary Fig. S5c,d respectively. Similarly for test case-0.4 and resonator thickness 0.6 µm, 0.8 µm, 1.0 µm, 1.2 µm, 1.4 µm scatter plots are shown in Supplementary Fig. S6d–f, S7c,d respectively.

### Angle of incidence

R^2^ scores obtained by trained 1D-CNN Regression models (Test Case-0.5) for assorted combinations of angle of incidence values with polynomial degree features are depicted using heat map in Fig. 6b. Highest R^2^ score of 0.9973, 0.9994, 0.9987, 1.0, 0.9979, 0.999, 0.9991, 0.9986, 0.9992 is achieved for angle of incidence value 0^0^, 10°, 20°, 30°, 40°, 50°, 60°, 70°, 80° respectively. Scatter plots of predicted absorption values by different 1D-CNN Regression models vs actual absorption values for test case-0.5 and angle of incidence value 0°, 10°, 20° are shown in Fig. 7g–i respectively.

R^2^ scores obtained by trained 1D-CNN Regression models for Test Case-0.4, Test Case-0.3, Test Case-0.2, and assorted combinations of angle of incidence values with polynomial degree features are depicted using heat map in Supplementary Fig. S4a–c. Scatter plots of predicted absorption values by different 1D-CNN Regression models vs actual absorption values for test case-0.5 and angle of incidence 30°, 40°, 50° are shown in Supplementary Fig. S5e–g respectively. Similarly for test case-0.4 and angle of incidence 0°, 10°, 20°, 30°, 40°, 50°, 60°, 70°, 80° scatter plots are shown in Supplementary Fig. S6g–i, S7e–g respectively.

## Conclusion

In conclusion, we have presented the two different graphene-metasurface absorber designs and obtained the high absorption for a broadband range including the ultraviolet and visible range. The O-shape design achieves absorption of 91% and 94% for the ultraviolet and visible range, respectively. We have also carried out the detailed analysis by varying the physical parameters such as resonator thickness and substrate thickness which slightly affects the absorption rate. The absorption response also remains unchanged for the wide angle of incidence. The response also remains the same with respect to the variation in graphene chemical potential.

It is demonstrated using experimental results that, 1D-CNN Regression is an efficient method for building the prediction model. It can predict absorption values of intermediate wavelengths with high accuracy (more than 0.999 R^2^ Score). It is also observed that high prediction accuracy is achieved in most of the cases when model is built using higher polynomial degree features.

## Supplementary Information

Supplementary Information.

## Data Availability

The data will be made available at a reasonable request to the corresponding author.

## References

[1] Huang Y, Liu L, Pu M, Li X, Ma X, Luo X (2018). A refractory metamaterial absorber for ultra-broadband, omnidirectional and polarization-independent absorption in the UV-NIR spectrum. Nanoscale.

[2] Rahmani M (2017). Nonlinear symmetry breaking in symmetric oligomers. ACS Photon..

[3] Liang C (2019). A broadband and polarization-independent metamaterial perfect absorber with monolayer Cr and Ti elliptical disks array. Results Phys..

[4] Li M (2019). Terahertz wideband perfect absorber based on open loop with cross nested structure. Results Phys..

[5] Qin F (2020). Ultra-broadband and wide-angle perfect solar absorber based on TiN nanodisk and Ti thin film structure. Sol. Energy Mater. Sol. Cells.

[6] Hatami M, Geng J, Jing D (2018). Enhanced efficiency in Concentrated Parabolic Solar Collector (CPSC) with a porous absorber tube filled with metal nanoparticle suspension. Green Energy Environ..

[7] Kildishev AV, Boltasseva A, Shalaev VM (2013). Planar photonics with metasurfaces. Science.

[8] Meinzer N, Barnes WL, Hooper IR (2014). Plasmonic meta-atoms and metasurfaces. Nat. Photon..

[9] Shaltout AM, Kildishev AV, Shalaev VM (2016). Evolution of photonic metasurfaces: From static to dynamic. J. Opt. Soc. Am. B.

[10] Li Y (2020). Semiconductor-nanoantenna-assisted solar absorber for ultra-broadband light trapping. Nanoscale Res. Lett..

[11] Zhang S, Park YS, Li J, Lu X, Zhang W, Zhang X (2009). Negative refractive index in chiral metamaterials. Phys. Rev. Lett..

[12] Sealy C (2018). Composite metamaterial bends the rules. Mater. Today.

[13] Ghasemi M, Choudhury PK, Baqir MA (2019). On the double nano-coned graphite metasurface-based multiband CIC absorber. Plasmonics.

[14] Sreekanth KV, Mahalakshmi P, Han S, Mani Rajan MS, Choudhury PK, Singh R (2019). Brewster mode-enhanced sensing with hyperbolic metamaterial. Adv. Opt. Mater..

[15] Te Lin K, Lin H, Yang T, Jia B (2020). Structured graphene metamaterial selective absorbers for high efficiency and omnidirectional solar thermal energy conversion. Nat. Commun..

[16] Yu P (2019). Broadband metamaterial absorbers. Adv. Opt. Mater..

[17] Zulkifli NAA (2020). Photocatalytic application of two-dimensional materials-based heterostructure based on molybdenum and tungsten disulfides and gallium nitride: A density-functional theory study. Mater. Today Commun..

[18] Jadeja R (2020). Numerical investigation of graphene-based efficient and broadband metasurface for terahertz solar absorber. J. Mater. Sci..

[19] Patel SK, Kosta YP (2014). Metamaterial superstrate-loaded meandered microstrip-based radiating structure for bandwidth enhancement. J. Mod. Opt..

[20] Ahir P, Patel SK, Parmar J, Katrodiya D (2019). Directive and tunable graphene based optical leaky wave radiating structure. Mater. Res. Express.

[21] Baqir MA (2019). Wide-band and wide-angle, visible- and near-infrared metamaterial-based absorber made of nanoholed tungsten thin film. Opt. Mater. Express.

[22] Zhang H, Yang J, Zhang H, Liu J (2018). Design of an ultra-broadband absorber based on plasma metamaterial and lumped resistors. Opt. Mater. Express.

[23] Geim AK, Novoselov KS (2007). The rise of graphene. Nat. Mater..

[24] Dideikin AT, Vul AY (2019). Graphene oxide and derivatives: The place in graphene family. Front. Phys..

[25] Tiwari SK, Sahoo S, Wang N, Huczko A (2020). Graphene research and their outputs: Status and prospect. J. Sci. Adv. Mater. Dev..

[26] Flynn GW (2015). Atomic scale imaging of the electronic structure and chemistry of graphene and its precursors on metal surfaces. Argonne IL (United States).

[27] Rifat AA, Rahmani M, Xu L, Miroshnichenko AE (2018). Hybrid metasurface based tunable near-perfect absorber and plasmonic sensor. Materials (Basel).

[28] Yu P (2019). A numerical research of wideband solar absorber based on refractory metal from visible to near infrared. Opt. Mater. (Amst).

[29] Patel SK, Charola S, Jani C, Ladumor M, Parmar J, Guo T (2019). Graphene-based highly efficient and broadband solar absorber. Opt. Mater. (Amst).

[30] Al-Rjoub A (2018). A design of selective solar absorber for high temperature applications. Sol. Energy.

[31] Wu D (2017). Numerical study of an ultra-broadband near-perfect solar absorber in the visible and near-infrared region. Opt. Lett..

[32] Liu Z, Liu G, Huang Z, Liu X, Fu G (2018). Ultra-broadband perfect solar absorber by an ultra-thin refractory titanium nitride meta-surface. Sol. Energy Mater. Sol. Cells.

[33] Bilal RMH, Baqir MA, Choudhury PK, Naveed MA, Ali MM, Rahim AA (2020). Ultrathin broadband metasurface-based absorber comprised of tungsten nanowires. Results Phys..

[34] Charola S, Patel SK, Dalsaniya K, Jadeja R, Nguyen TK, Dhasarathan V (2021). Numerical investigation of wideband L-shaped metasurface based solar absorber for visible and ultraviolet region. Phys. B Condens. Matter.

[35] Petrone N (2012). Chemical vapor deposition-derived graphene with electrical performance of exfoliated graphene. Nano Lett..

[36] Novoselov KS (2005). Two-dimensional atomic crystals. Proc. Natl. Acad. Sci..

[37] Zou T (2020). High-speed femtosecond laser plasmonic lithography and reduction of graphene oxide for anisotropic photoresponse. Light Sci. Appl..

[38] Yu P (2020). Ultra-wideband solar absorber based on refractory titanium metal. Renew. Energy.

[39] Patel SK, Charola S, Parmar J, Ladumor M (2019). Broadband metasurface solar absorber in the visible and near-infrared region. Mater. Res. Express.

[40] Lin H (2019). A 90-nm-thick graphene metamaterial for strong and extremely broadband absorption of unpolarized light. Nat. Photonics.

[41] Katrodiya D, Jani C, Sorathiya V, Patel SK (2019). Metasurface based broadband solar absorber. Opt. Mater. (Amst).

[42] Azad AK (2016). Metasurface broadband solar absorber. Sci. Rep..

[43] Patel SK, Charola S, Parmar J, Ladumor M, Ngo QM, Dhasarathan V (2020). Broadband and efficient graphene solar absorber using periodical array of C-shaped metasurface. Opt. Quantum Electron..

[44] Liu B (2018). Multiband and broadband absorption enhancement of monolayer graphene at optical frequencies from multiple magnetic dipole resonances in metamaterials. Nanoscale Res. Lett..

[45] Sang T, Gao J, Yin X, Qi H, Wang L, Jiao H (2019). Angle-insensitive broadband absorption enhancement of graphene using a multi-grooved metasurface. Nanoscale Res. Lett..

[46] Rufangura P, Sabah C (2017). Graphene-based wideband metamaterial absorber for solar cells application. J. Nanophoton..

[47] Babu, G. S., Zhao, P., & Li, X. L. Deep convolutional neural network based regression approach for estimation of remaining useful life. In *Lecture Notes in Computer Science (Including Subseries Lecture Notes in Artificial Intelligence and Lecture Notes in Bioinformatics)*. 10.1007/978-3-319-32025-0_14 (2016).

