# Supplementary material for: Graphene-based metasurface solar absorber design with absorption prediction using machine learning
Source: Sci Rep. 2022 Feb 16;12:2609. doi: 10.1038/s41598-022-06687-6 (PMC8850562; doi:10.1038/s41598-022-06687-6)
Supplement: Supplementary file 1 — Supplementary Information. [file 41598_2022_6687_MOESM1_ESM.docx]

**Supplementary Material**

**Graphene-based metasurface solar absorber design with absorption prediction using machine learning**

Juveriya Parmar^1^,Shobhit K. Patel^1,2,*^ and Vijay Katkar^2^

^1^Department of Electronics and Communication, Marwadi University, Rajkot-360003, India

^2^Department of Computer Engineering, Marwadi University, Rajkot-360003, India

*Corresponding Author: shobhitkumar.patel@marwadieducation.edu.in

Correlation analysis is the most widely used approach to discover the existence of a relationship between two variables. Equation (S1) is used to calculate joint variability (covariance) between two variables.

| $Cov(X, Y) =\frac{\sum_{i=i}^{N} (x_{i}- \bar{X})*(y_{i}-\bar{Y})}{N}$ | (S1) |
| --- | --- |

Pearson’s correlation coefficient (ρ) is calculated by normalizing the covariance value. It is normalized using equation (S2).

| $\rho(X,Y) =\frac{Cov(X, Y)}{\sigma X*\sigma Y}$ | (S2) |
| --- | --- |

Spearman’s correlation coefficient, also known as Pearson’s correlation coefficient between the rank variables is calculated using equation (S3).

| $\rho R(X),R(Y) =\frac{Cov(R(X), R(Y))}{\sigma R(X)*\sigma R(Y)}$ | (S3) |
| --- | --- |

Pearson’s correlation coefficient and Spearman’s correlation coefficient are used to measure the relationship between two variables. A value close to +1 or -1 is considered a good relationship.

Covariance, Pearson’s Correlation, Spearman’s Correlation between wavelength values and absorption values for assorted Angles of Incidence values, Resonator thickness values, Substrate thickness is shown in Fig. S1.

It can be observed that these values are very low and close to zero. As a result, 1D-CNN regression models need to be trained using higher degree polynomial features. Nth Degree polynomial feature value is calculated using equation (S4).

| $f\left( x \right)=a_{n}x^{n}+ a_{n-1}x^{n-1}+ a_{n-2}x^{n-2}+\ldots+ a_{0}x^{0}$ | (S4) |
| --- | --- |


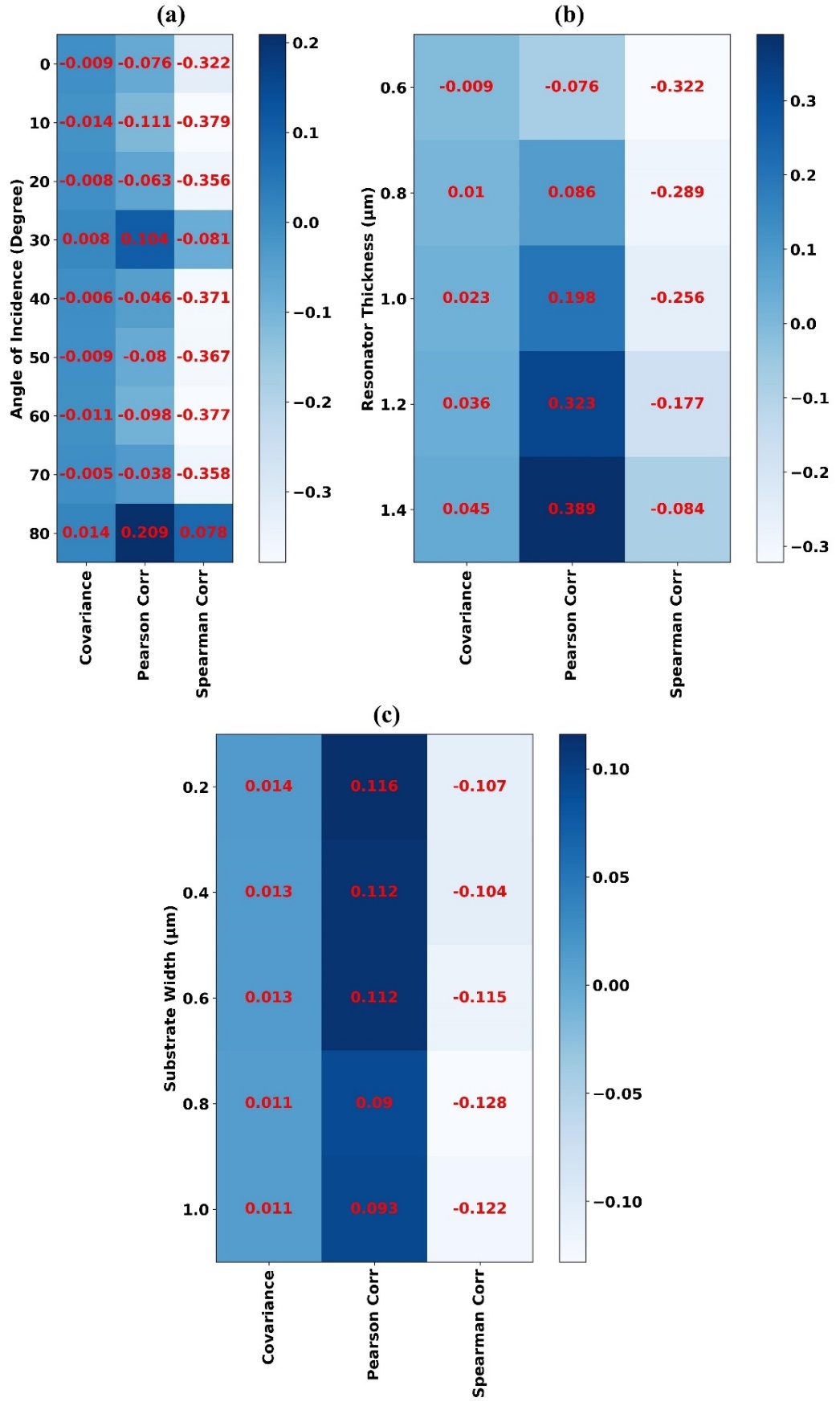


**Fig. S1** Heatmap of Covariance, Pearson’s Correlation, Spearman’s Correlation between wavelength values and absorption values for assorted (a) Angles of Incidence (b) Resonator thickness values, (c) substrate thickness

R^2^ scores obtained by trained 1D-CNN Regression models for Test Case-0.4, Test Case-0.3, Test Case-0.2, and assorted combinations of substrate thickness values with polynomial degree features are depicted using heat map in supplementary Fig. S2 (a-c) respectively.


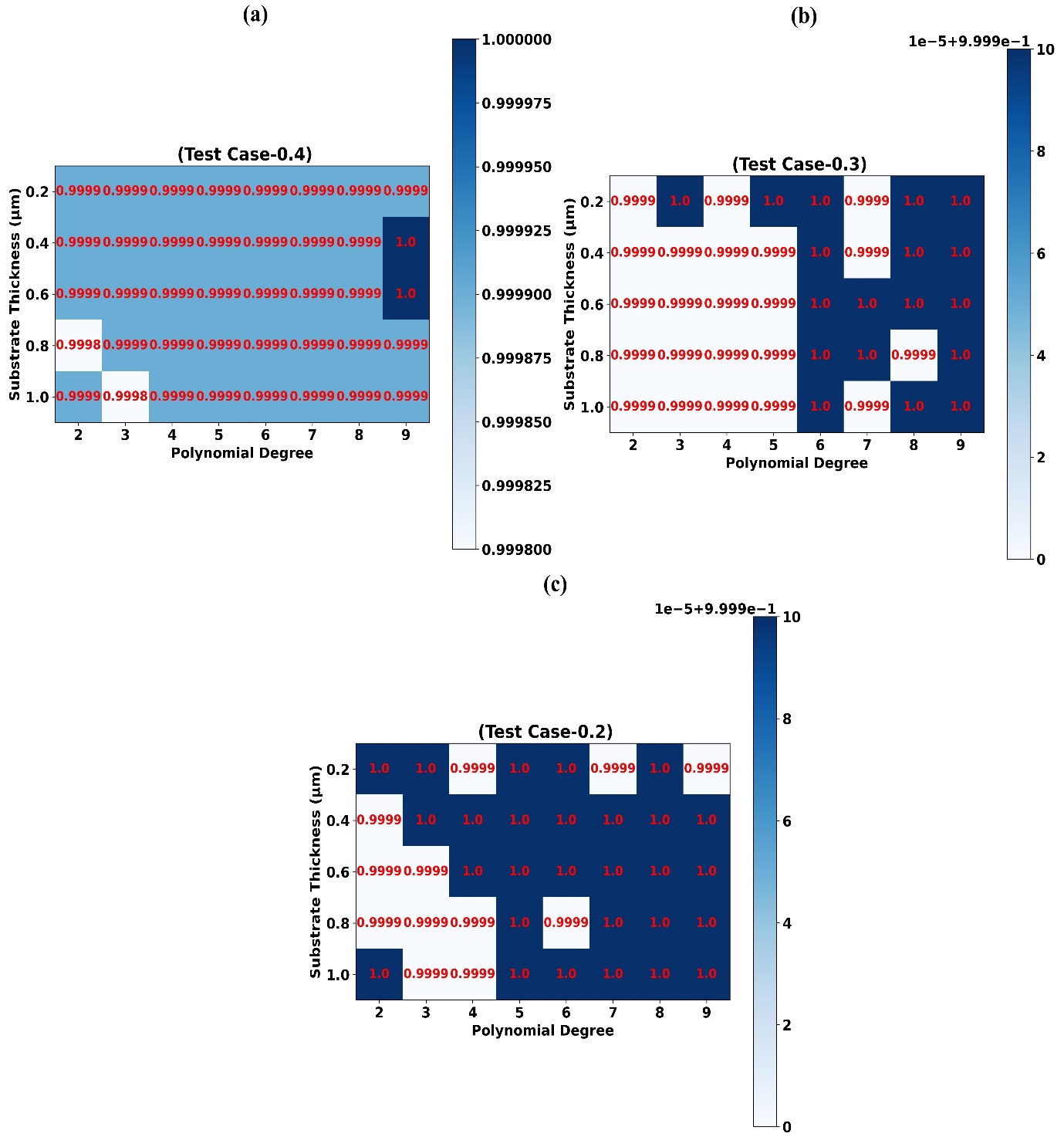


**Fig. S2** Prediction accuracy (R^2^ Score) of 1D-CNN Regressor models trained for assorted values of (a) Substrate Thickness and Polynomial Degree features (Test Case C-0.4) (b) Substrate Thickness and Polynomial Degree features (Test Case C-0.3) (c) Substrate Thickness and Polynomial Degree features (Test Case C-0.2)

R^2^ scores obtained by trained 1D-CNN Regression models for Test Case-0.4, Test Case-0.3, Test Case-0.2, and assorted combinations of resonator thickness values with polynomial degree features are depicted using heat map in supplementary Fig. S3 (a-c) respectively.


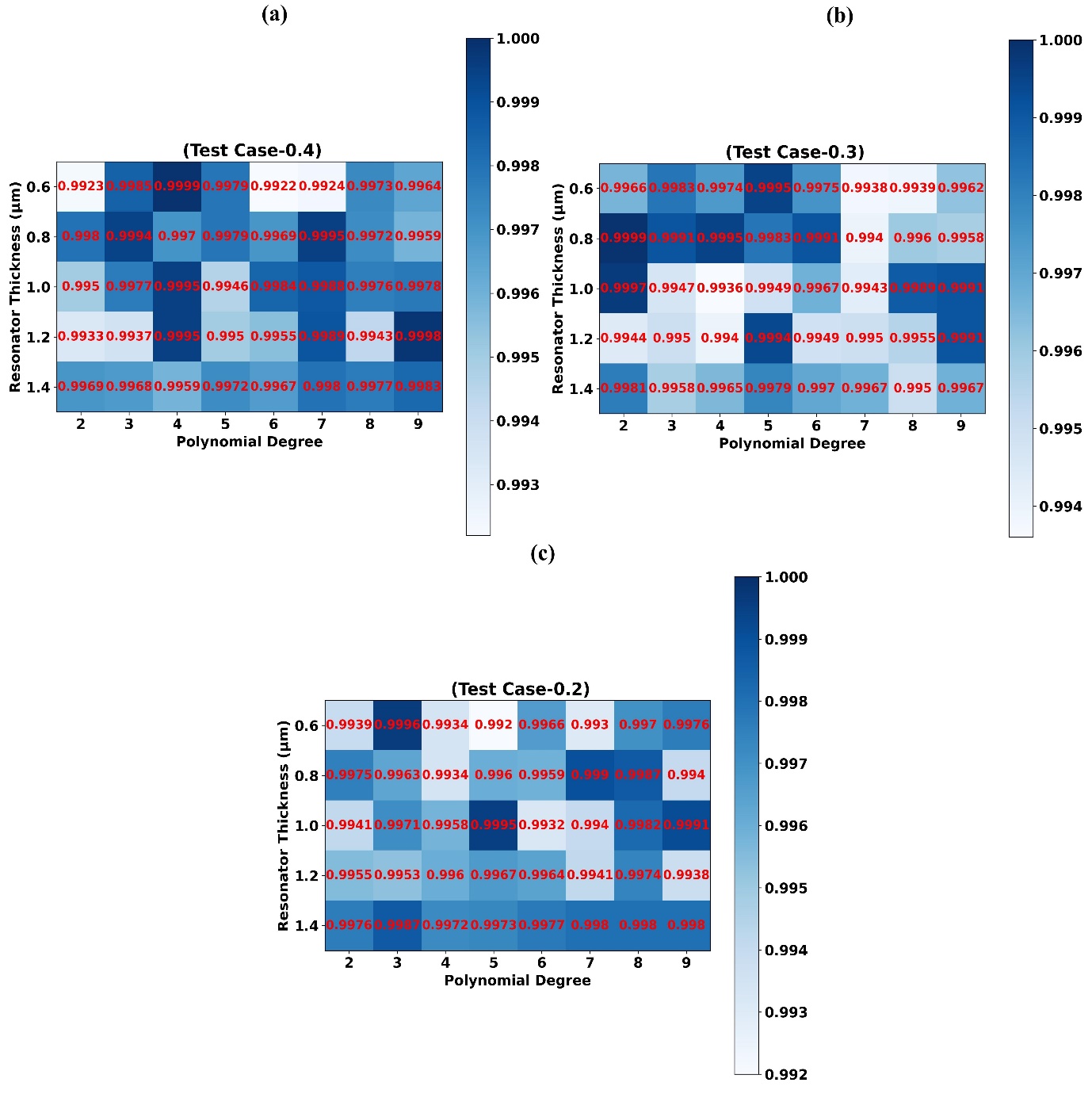


**Fig. S3** Prediction accuracy (R^2^ Score) of 1D-CNN Regressor models trained for assorted values of (a) Resonator Thickness and Polynomial Degree features (Test Case C-0.4) (b) Resonator Thickness and Polynomial Degree features (Test Case C-0.3) (c) Resonator Thickness and Polynomial Degree features (Test Case C-0.2)

R^2^ scores obtained by trained 1D-CNN Regression models for Test Case-0.4, Test Case-0.3, Test Case-0.2, and assorted combinations of angle of incidence values with polynomial degree features are depicted using heat map in supplementary Fig. S2 (a-c) respectively.


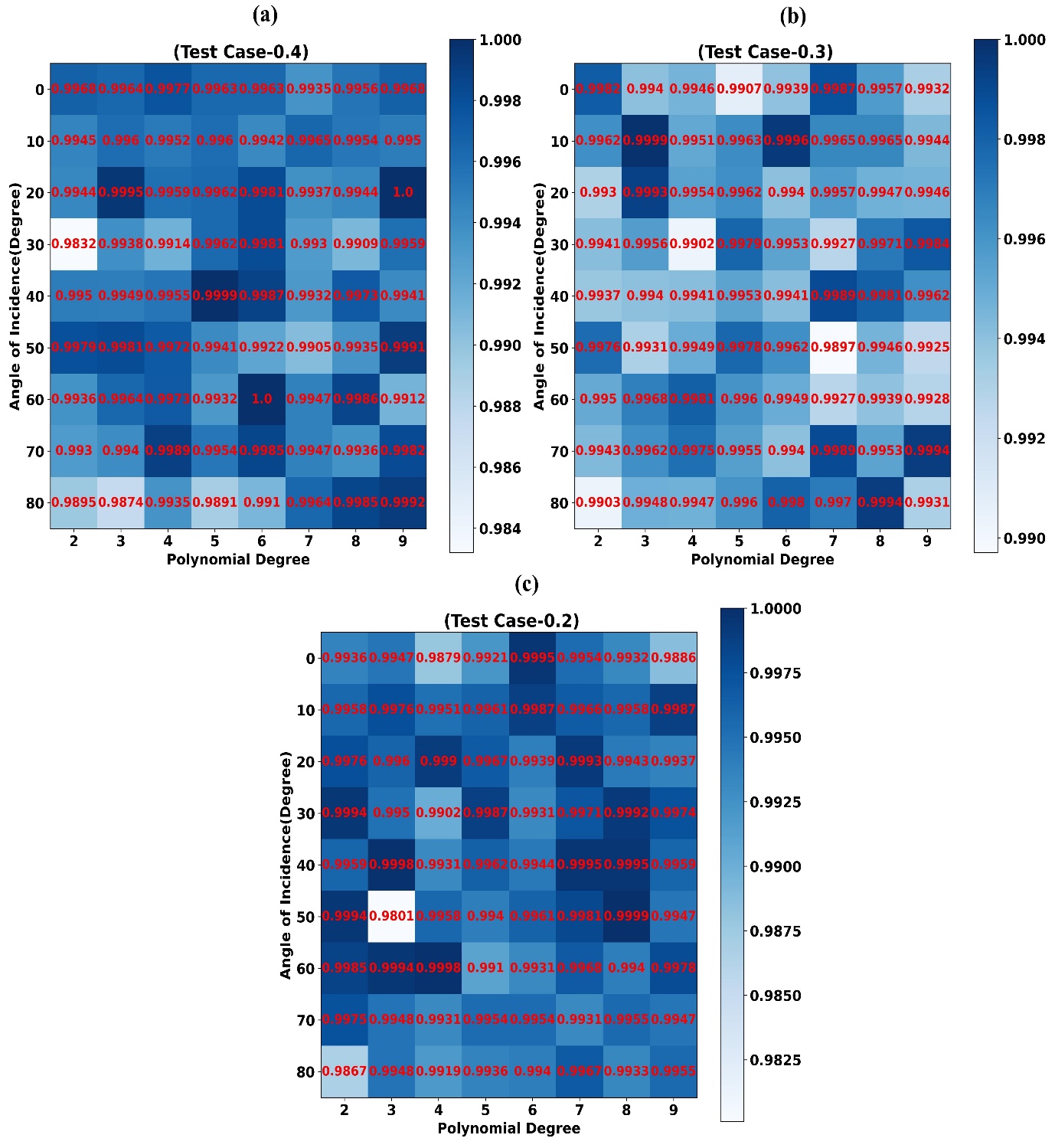


**Fig. S4** Prediction accuracy (R^2^ Score) of 1D-CNN Regressor models trained for assorted values of (a) Angle of Incidence and Polynomial Degree features (Test Case C-0.4) (b) Angle of Incidence and Polynomial Degree features (Test Case C-0.3) (c) Angle of Incidence and Polynomial Degree features (Test Case C-0.2)

Scatter plots of predicted absorption values by different 1D-CNN Regression models vs actual absorption values for test case-0.5 and substrate thickness 0.8 µm, 1.0 µm are shown in supplementary Fig. S5 (a-b) respectively. Similarly for test case-0.4 and substrate thickness 0.2 µm, 0.4 µm, 0.6 µm, 0.8 µm, 1.0 µm scatter plots are shown in supplementary Fig. S6 (a-c), Fig S7 (a-b) respectively.

Scatter plots of predicted absorption values by different 1D-CNN Regression models vs actual absorption values for test case-0.5 and resonator thickness 1.2 µm, 1.4 µm are shown in supplementary Fig. S5 (c-d) respectively. Similarly for test case-0.4 and resonator thickness 0.6 µm, 0.8 µm, 1.0 µm, 1.2 µm, 1.4 µm scatter plots are shown in supplementary Fig. S6 (d-f), Fig S7 (c-d) respectively.

Scatter plots of predicted absorption values by different 1D-CNN Regression models vs actual absorption values for test case-0.5 and angle of incidence 30^0^, 40^0^, 50^0^ are shown in supplementary Fig. S5 (e-g) respectively. Similarly for test case-0.4 and angle of incidence 0^0^, 10^0^, 20^0^, 30^0^, 40^0^, 50^0^, 60^0^, 70^0^, 80^0^ scatter plots are shown in supplementary Fig. S6 (g-i), Fig S7 (e-g) respectively.


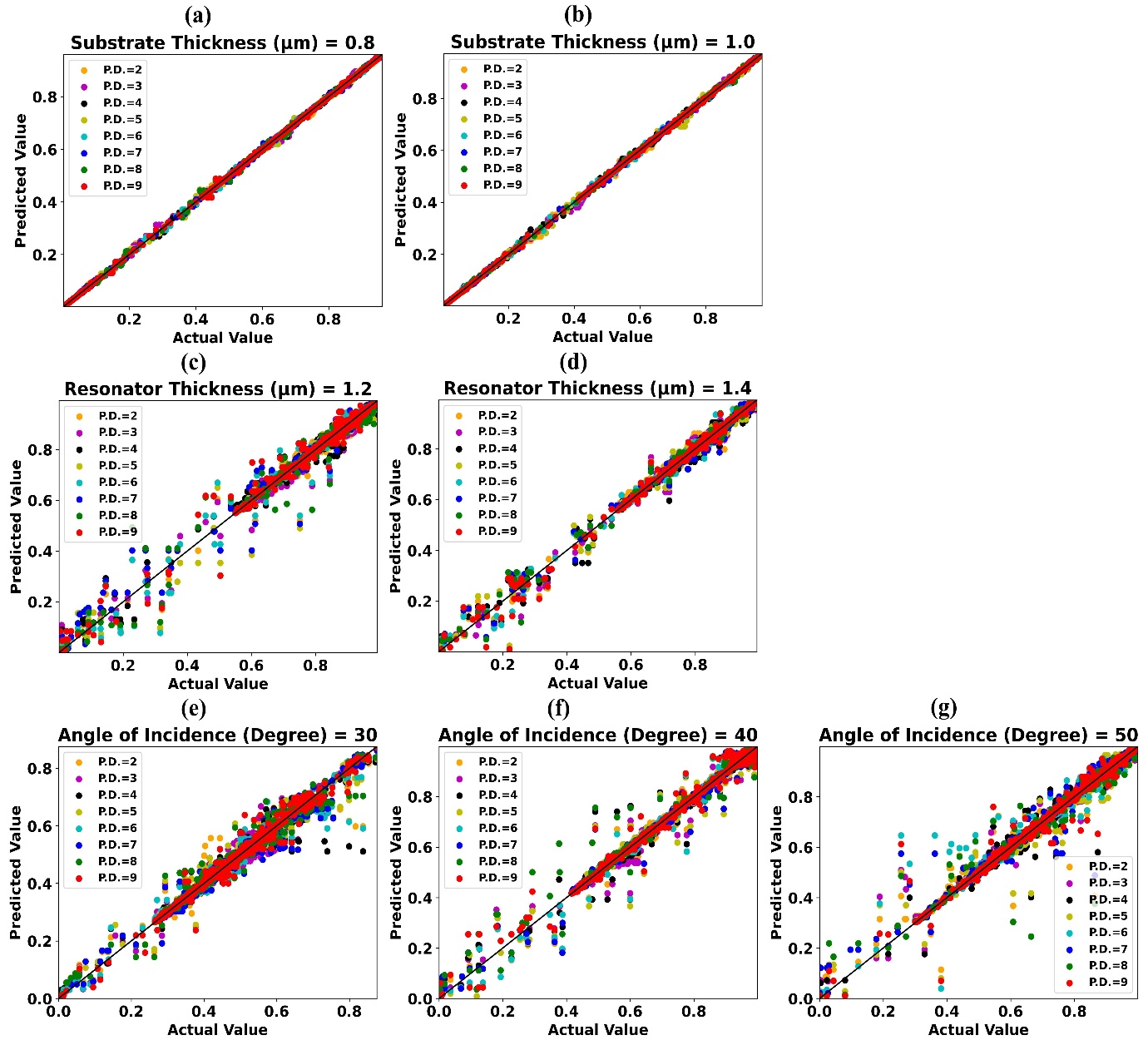


**Fig. S5** Predicted absorption value by different 1D CNN Regressors vs Actual absorption value for test case C-0.5 and (a) Substrate Thickness = 0.8µm (b) Substrate Thickness = 1.0µm (c) Resonator Thickness=1.2µm (d) Resonator Thickness = 1.4µm (e) Angle of Incidence = 30^0^ (f) Angle of Incidence = 40^0^ (g) Angle of Incidence = 50^0^


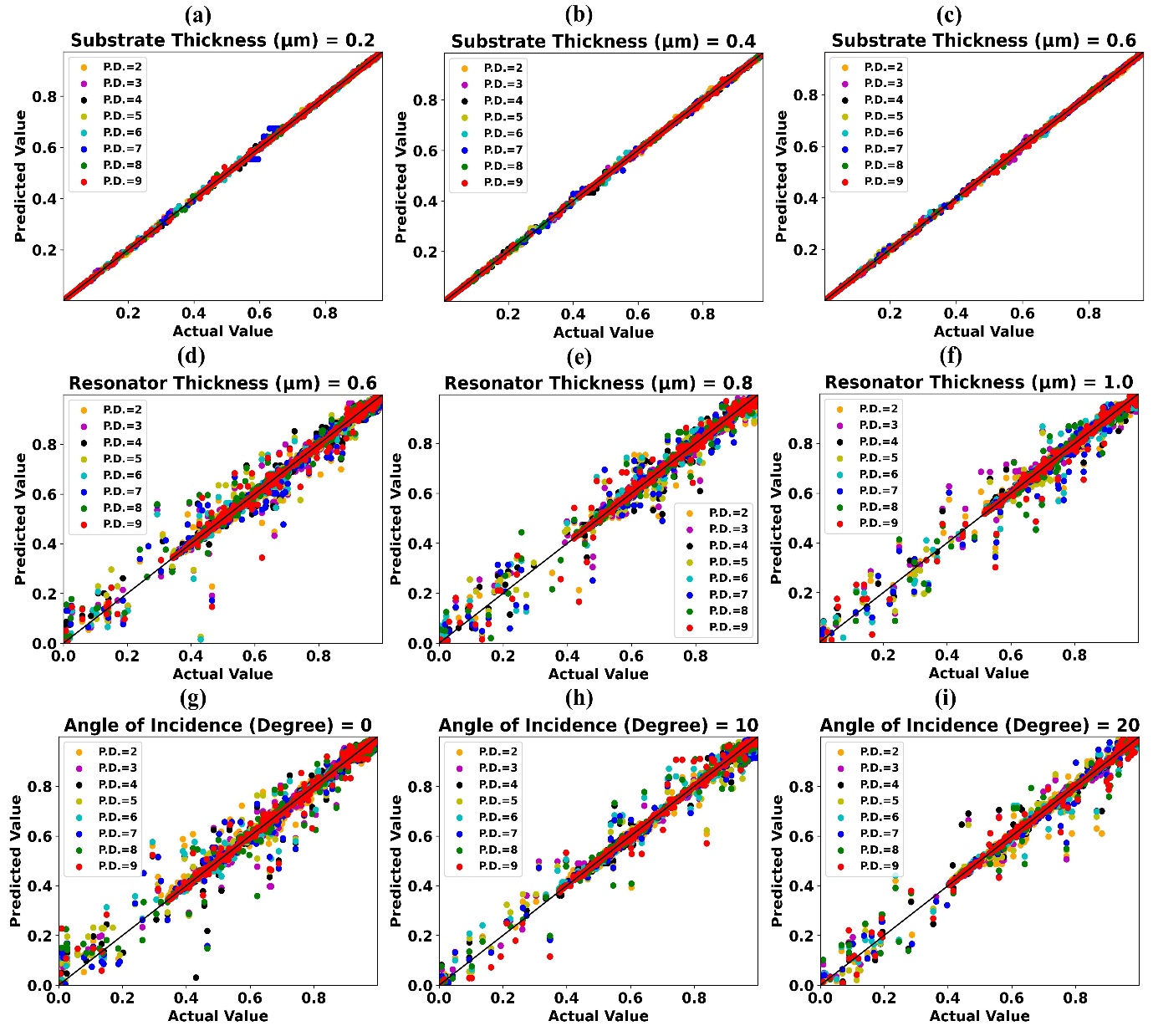


**Fig. S6** Predicted absorption value by different 1D CNN Regressors vs Actual absorption value for test case C-0.4 and (a) Substrate Thickness = 0.2µm (b) Substrate Thickness = 0.4µm (c) Substrate Thickness = 0.6µm (d) Resonator Thickness=0.6µm (e) Resonator Thickness = 0.8µm (f) Resonator Thickness = 1.0µm (g) Angle of Incidence = 0^0^ (h) Angle of Incidence = 10^0^ (i) Angle of Incidence = 20^0^


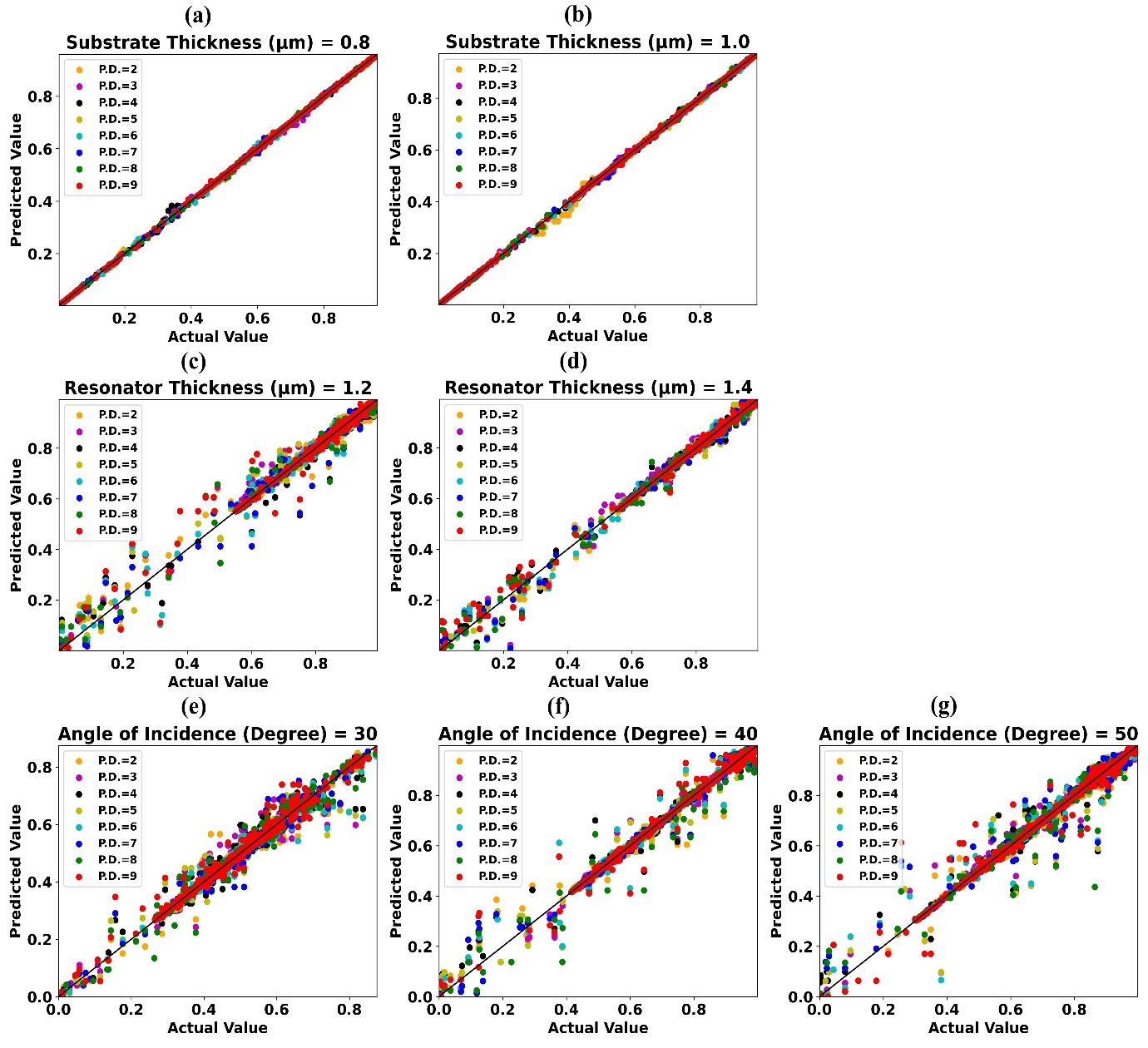


**Fig. S7** Predicted absorption value by different 1D CNN Regressors vs Actual absorption value for test case C-0.4 and (a) Substrate Thickness = 0.8µm (b) Substrate Thickness = 1.0µm (c) Resonator Thickness=1.2µm (d) Resonator Thickness = 1.4µm (e) Angle of Incidence = 30^0^ (f) Angle of Incidence = 40^0^ (g) Angle of Incidence = 50^0^
